# Barriers to Care for Adults With Sickle Cell Disease: A Qualitative Descriptive Study

**DOI:** 10.1111/hex.70310

**Published:** 2025-05-24

**Authors:** Chanell Grismore, Lisa R. Roberts, Zephon D. Lister, Akshat Jain, Julio Silvestre, Ja'Nece Dickerson, Susanne B. Montgomery

**Affiliations:** ^1^ School of Behavioral Health Loma Linda University Loma Linda California USA; ^2^ School of Nursing Loma Linda University Loma Linda California USA; ^3^ Loma Linda University School of Medicine Division of Hematology Oncology Loma Linda California USA; ^4^ Department of Supportive Care Medicine Loma Linda University Hospital Loma Linda California USA; ^5^ Loma Linda University Hospital Loma Linda California USA

**Keywords:** health services accessibility, sickle cell, social determinants of health, stereotyping, transitional care

## Abstract

**Objective:**

We sought to understand barriers to care among adults with sickle cell disease (SCD) within our healthcare system.

**Study Setting and Design:**

This qualitative descriptive study was conducted as part of a needs and assets assessment in preparation for the development of an adult sickle cell clinic.

**Data Sources and Analytic Sample:**

We conducted key informant interviews (*n* = 19) and a focus group (*n* = 10) with administrators, patients, caregivers and healthcare providers (HCPs). Audio recordings were transcribed verbatim, coded inductively and analysed thematically.

**Principle Findings:**

Four themes emerged: (1) People with SCD identified structural barriers, such as the lack of individualised care, access barriers to medical, community, adult‐specific resources and inadequate transitional care support from paediatric to adult care. (2) HCP‐related barriers, which included a lack of understanding and education, communication gaps and access barriers to finding HCPs trained in caring for people with SCD. (3) Discrimination biases by HCPs and outright prejudice towards people with SCD. (4) Financial barriers such as lack of incentives for comprehensive SCD centres and HCPs and funding reimbursement. We found that adult people with SCD had unique challenges. Interestingly, patients had perspectives similar to HCPs, while administrators were more focused on the financial barriers that affect continuity of care.

**Conclusions:**

These barriers require a concerted and multidisciplinary effort from patients, caregivers, HCPs, administrators and the community.

**Patient or Public Contribution:**

Patients, caregivers and community members played a crucial role in this study by sharing their lived experiences and perspectives on barriers to care for adults with SCD.

## 1 Introduction

Sickle cell disease (SCD), a complex, chronic condition, is the most common inherited blood disorder in the United States [1], though classified as a rare disease [2, 3]. SCD can affect individuals of all ethnic backgrounds, but is most common among people of African, Hispanic, Middle Eastern and South East Asian descent [4]. SCD affects millions of people globally [5], and approximately 100,000 people in the United States, most of them are African American/Black [1].

While SCD comes with critical needs everywhere, even in a high‐resource environment like the United States, it has resulted in a uniquely medically underserved population, given its complexity and who it mostly affects [6]. Disparities in SCD funding, treatment, research and outcomes are well documented [3, 7, 8, 9]. Additionally, despite advances in treatment, SCD contributes to all‐cause mortality and premature death (median life expectancy 60 years compared to 75 years for people without SCD) [10]. The disability‐adjusted life‐year (DALY) burden of SCD on individuals is severe, with pain as a key clinical manifestation among patients with SCD, who experience poor health‐related quality of life (HRQOL) [8, 11, 12]. Since pain and morbidity span the life course of people living with SCD [12], it presents with devastating effects on multiple domains of health (physical, mental, social and spiritual). Furthermore, individuals with SCD experience disproportionate detrimental social determinants of health, which result in clinical barriers and worsened outcomes [13].

Individuals with SCD suffer from lifelong haemolysis, leading to hypoxaemia and hyperinflammation. This leads to multiple organ toxicities presenting most commonly as vaso‐occlusive pain crises and recurrent organ damage due to vaso‐occlusive‐related tissue ischaemia. Anaemia, acute chest syndrome, stroke, splenic sequestration, perinatal mortality and other complications to physical health are common [4]. Common delays in care among underrepresented minorities further exacerbate physical health symptoms and worsen outcomes [13]. Often not talked about, but not to be overlooked, additional physical complications such as delayed growth and sexual maturation and priapism and infertility, correlate negatively with mental, social and spiritual health domains among people with SCD.

Living with a painful condition like SCD throughout life can have significant psychological impacts. Patients with SCD often experience comorbidities such as anxiety, depression, stress, cognitive impairment and risk for post‐traumatic stress disorder (PTSD) due to the unpredictable nature of the disease, frequent hospitalisations, chronic pain and limitations in daily activities [11, 12, 14]. All too often, people with SCD face stigmatisation within healthcare and detrimental social determinants of health, including discrimination‐related stress, which further impacts their health [15]. Barriers to care include insufficient access to specialised SCD care, a shortage of certified haematologists with experience in SCD management, sensitisation of the primary care providers, decreased access to pain management due to the opioid crisis in the United States, underutilisation of existing treatment regimens for SCD, and healthcare systems requiring transition of care [3, 15].

Due to these many complexities, SCD often affects patients' social relationships and interactions. They often experience stigma and discrimination in healthcare settings, further contributing to health disparities and inequities [12, 13]. Misconceptions within the general public related to the disease may lead to social isolation, difficulties in forming and maintaining friendships and romantic relationships [16], and challenges in educational and work settings due to absenteeism [8, 9, 11].

Not surprisingly, SCD can prompt existential questions, spiritual distress and challenges or changes to one's faith or belief system [16]. Conversely, spirituality can also provide strength, hope and a sense of meaning and purpose in the face of adversity [17] and thus, for many, plays a significant role in coping with this complicated, all‐consuming chronic illness.

Comprehensive care for people with SCD should address these multiple domains of health, including medical management to reduce morbidity and early mortality, mental health support, social services and spiritual care, to improve their well‐being and maximise quality of life [4]. Models of care that include these domains are the Classic Comprehensive model of having a medical family home, which includes a dedicated clinical space and dedicated clinical staff [18].

Given advances in care, especially in the generally better coordinated paediatric care, patient outcomes keep improving, but coordinated adult care options are often lagging. Especially when patients with SCD transition from paediatric to adult care, they are particularly vulnerable as they must see new healthcare providers (HCPs), take on the responsibility of making and attending multiple appointments in various settings and manage their daily self‐care needs [19]. Emergency department utilisation increases dramatically during this time frame [20]. Disparities in outcomes become more pronounced in adult SCD patients after they transition from paediatric care [8, 20]. Therefore, we sought to understand barriers to care among adults with SCD by listening to the voices of key informants and participants of SCD care within our healthcare system.

## 2 Materials and Methods

This qualitative descriptive study was part of a comprehensive needs and assets assessment in preparation for the development of an adult sickle cell clinic for a comprehensive health system in Southern California, which serves increasing numbers of SCD patients and seeks to develop a much‐needed medical home for patients with SCD. After obtaining Institutional Review Board approval (#5190109, 28 December 2021), purposive sampling techniques were utilised to recruit adults with SCD, caregivers, healthcare administrators and providers caring for patients with SCD, and community stakeholders. Adults (≥ 18 years) with a confirmed SCD diagnosis who currently or previously received SCD care in the Inland Empire were recruited through clinics, outreach events, personal referrals and support groups. Administrators, advocates and providers were emailed if they served the region as haematology, primary/family/internal/palliative‐care clinicians, ancillary staff (social work, psychology and child life) or leaders of health systems, health plans or SCD community‐based organisations. Individuals outside these roles or practising beyond the Inland Empire were excluded.

Informed consent was obtained before data collection. Four doctoral‐level, qualitatively trained research study personnel associated with the adult sickle cell centre completed data collection from January 2022 to April 2023, using a semi‐structured interview guide. In total, 19 key informant interviews and 1 feedback focus group (*n* = 10) were conducted. On average, the interviews were 45 min in length, and the focus group was 1 h and 30 min. The key informant interviews were conducted virtually, and the focus group was done in person in April 2023, observing pandemic precautions. Although the California stay‐at‐home order was rescinded on 25 January 2021 and Covid‐19 rates were among the lowest in the nation, given the vulnerability of our sample population, the following precautions were taken: (1) on‐site COVID‐19 testing, (2) masks provided and (3) social distancing observed. All audio recordings were transcribed verbatim, and any identifiable information was redacted. Initial interviews were coded inductively by two individuals to develop a codebook. Coding of all transcripts proceeded using the codebook in NVivo software, and data were analysed iteratively using Charmaz's approach to categorise the codes [21]. Finally, we conducted thematic analysis to describe and interpret patterns of the phenomenon.

## 3 Findings

### 3.1 Participant Characteristics

Nearly half (45%) of the participants were patients receiving treatment for SCD (age 25–57 years), and their caregivers, at different centres located in Riverside, San Bernardino and Los Angeles Counties. There were an even number of male and female patients (six each), and one who identified as non‐binary/third gender. Key informant interviews were also conducted with health system leaders and senior executives. At 21% of all participants, HCPs constituted the second largest category and included providers specialising in areas such as palliative care, internal medicine, haematology and family medicine. Healthcare administrators accounted for 17% of the total participants. Advocates accounted for 14% of the total participants. Among the advocates were prominent opinion leaders from the community and community health professionals. One participant (3%) came from a payor group.

An overview of the participants is provided in Table 1.

**Table 1:** Participants (*N* = 29).

Characteristics	*n* (%)	Participation activity
Healthcare administrator	5 (17%)	Key informant interview
Patients and caregivers	13 (45%)	Focus group discussion or key informant interview
Healthcare provider (HCP)^a^	6 (21%)	Key informant interview
Payor group	1 (3%)	Key informant interview
Advocates^b^	4 (14%)	Key informant interview

^a^ Physicians, nurses and social workers.

^b^ Patient advocate and community health workers.

### 3.2 Description of Themes

**Figure 1:**
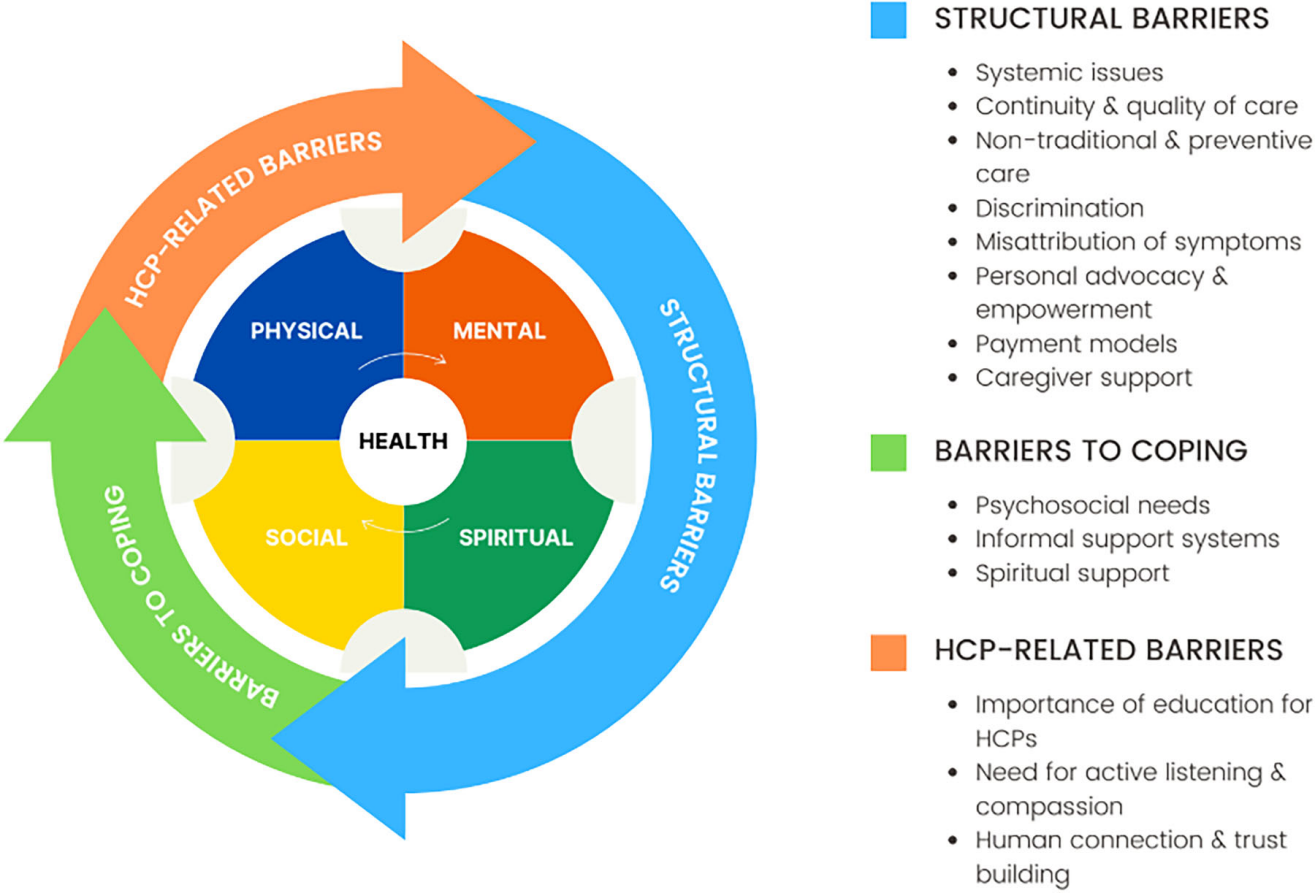
Barriers to care for sickle cell patients, impacting physical, mental, social and spiritual health.

The participants' overarching concern was that there are numerous interrelated barriers to care for the Sickle Cell community. In examining the data, three primary themes emerged: (1) structural barriers, (2) HCP‐related barriers and (3) barriers to coping. A visual representation of barriers is provided in Figure 1. While there is some overlap, each theme elucidated a unique dimension of barriers to care for the sickle cell community. Data highlights the role that systemic racism plays in the structural barriers faced by the Sickle Cell community when accessing comprehensive adult Sickle Cell care. Findings also identified the need for knowledgeable, compassionate providers and the need to address the psychosocial needs of patients to facilitate receptivity to care, considering multiple discouraging experiences. These themes are further elaborated upon in the sections below, supported by illustrative quotes from participants.

#### 3.2.1 Theme 1: Structural Barriers

Structural barriers are seen as closely tied to the manifestation of systemic racism related to the illness, affecting patients' access to care and healthcare experiences. Participants identified structural barriers as organisational and systemic limitations that hinder sickle cell patients from accessing comprehensive care. These barriers include the limited availability or complete absence of needed specialised medical service (i.e., specialist in haematology/oncology and pain management), inadequate healthcare infrastructure, inconsistencies in care across different healthcare settings (e.g., outpatient vs. inpatient care or across different health systems), and prolonged waiting times in emergency rooms and urgent care facilities. One participant discussed how resources and personnel are negatively impacted by the lack of reimbursement for services.

‘… the lack of funding and reimbursement for providing adequate care, I think is a huge gap in the industry as a whole.’

Participant 29, administrator

‘And so not only is there reimbursement not there, but we're financially penalized where we're having money taken from us because of higher readmission rates among other things.’

Participant 29, administrator

From the payor and healthcare system administration's perspective, SCD care is a conundrum financially. Providing high‐quality care to people with SCD is costly and inadequately reimbursed, yet insufficient care results in frequent readmissions and poorer outcomes, which increase costs and incur penalties.

Other participants pointed to the pervasive impact of systemic racial discrimination, which compounds these structural challenges. They described biases in medical treatment, inequitable resource allocation and discriminatory practices within the healthcare industry as significant obstacles to equitable care.

In addition to these already significant structural barriers, another major concern highlighted was the pervasive stigma associated with SCD. A HCP living with SCD shared their experience, stating,

‘There's a lot of stigma with the disease, even as a resident when people still didn't know that I had the disease. I would hear very negative comments coming from other residents or other physicians and attendings themselves.’

Participant 16, HCP

This quote underscores the widespread negative attitudes and stigma within the medical community. Even among healthcare professionals, there are entrenched prejudices that affect how patients with SCD are perceived and treated. Such stigma can lead to a lack of empathy and a reduction in the quality of care provided, as the negative perceptions influence how patients' needs and symptoms are addressed.

Participants also reported discrimination and bias, particularly regarding the intersection of race and chronic illness. One patient highlighted the compounded challenges of being African American with SCD, noting,

‘There is a stigma associated with us being African‐American and as adults when we walk in the door, when they walk in the door, both categories, they're perceived with the prejudice. With the historic prejudice.’

Participant 26, patient

This statement reflects how racial and historical prejudices collide with the experience of living with SCD, creating at times overwhelming barriers to adequate medical care. The dual impact of racial bias and the stigmatisation of SCD can result in patients facing prejudiced treatment and a lack of appropriate medical attention.

Furthermore, participants expressed frustration with the lack of personalised care for patients with SCD. One patient articulated this concern by saying,

‘They try to treat all [people with SCD/T] the same, but that's not how it works.’

Participant 17, patient

This comment frames the system's preference to identify standardised approaches to treating SCD, which is nearly impossible given the complexity of the illness and is in direct contrast to trying to provide much‐needed, comprehensive, individualised care to patients. Such a one‐size‐fits‐all approach can overlook the unique challenges faced by each patient, leading to inadequate or inappropriate care. Indeed, there are different SCD genotypes that some HCPs may not be familiar with, and these types differ in presentation and need an optimal management profile. Haemoglobin SS, S‐Beta zero and SC are the most prevalent sickle cell subtypes and typically manifest with progressive severity. While other subtypes (SB+, SD, SE and SO) are often less severe, sickle cell trait may be, but is not always, asymptomatic [22]. In other words, disease manifestation is highly individual and is highly affected by the length of disease exposure (age), type and past lack of comprehensive treatment. Thus, the lack of individualised treatment can exacerbate the difficulties experienced by patients and hinder their ability to manage their condition effectively.

Participants revealed a complex interplay of systemic racism, bias and structural shortcomings in the care of patients with SCD. Coupled with the debilitating nature of the disease, these structural issues lead to poor outcomes. Addressing these systemic challenges requires confronting stigma and prejudice within the medical community while also prioritising the development of personalised, patient‐centred care approaches. However, participants also emphasised that these efforts are often undermined by HCPs' lack of preparedness to treat this complex disease, compassion and understanding of the multiple aspects that are part of SCD. This gap in provider competence and empathy compounds the structural barriers, further diminishing the quality of care and patient outcomes. Bridging these gaps is essential to improving not only access to care but also the experiences and trust of individuals living with SCD. The next theme explores how HCP‐related barriers, such as insufficient awareness, knowledge and sensitivity, further exacerbate the challenges faced by SCD patients.

#### 3.2.2 Theme 2: HCP‐Related Barriers

The knowledge gap and barriers to understanding and compassion from healthcare teams impact both the availability and quality of care. While providers also acknowledged this issue, patients contributed most to the next category of barriers, namely HCP‐related barriers, which encompass obstacles arising from interactions with HCPs. Participants highlighted several critical issues, including the lack of specialised care, which results in a lack of much‐needed specialised treatments, challenges faced during medical crises and significant resource constraints that hinder the availability and quality of care. Additional issues identified were insufficient understanding of SCD among HCPs, compounded by inadequate education and training for all levels of providers, including nurses, aids and administrative front desk staff. Participants also described communication gaps and the difficulty of finding HCPs who are both adequately trained and willing to provide comprehensive care for individuals with SCD. These barriers not only impact the timeliness and effectiveness of care but also erode trust and confidence in the healthcare system, exacerbating the challenges faced by patients living with SCD.

A participant noted that ‘*some hematologists don't even deal with sickle cell*’ (Participant 26, advocate), pointing to a significant gap in the availability of medical professionals who have specific expertise in this condition. This lack of specialised knowledge is crucial because SCD requires a nuanced understanding of its chronic nature and the specific medical interventions needed. Participants highlighted that the absence of haematologists who are familiar with sickle cell can lead to inadequate treatment plans and suboptimal management of the disease. Without access to specialists who are well‐versed in SCD, patients may not receive the comprehensive care necessary to manage their condition effectively. This shortfall in specialised care can contribute to a broader problem where patients with SCD, a complex, chronic condition, struggle to get the expert attention required for their unique health needs.

In addition to the shortage of specialised care, patients with SCD frequently face challenges during medical emergencies. One participant described these difficulties, stating,

‘Our clients are in a crisis or they are in the emergency room and the doctors are like, I'm not here to cure sickle cell. You just need to get out.’

Participant 27, HCP

Patients identified that whenever seeking care for severe pain or complications, they are met with dismissive or unhelpful responses from HCPs. The lack of willingness or capability of some emergency room doctors to manage sickle cell crises can exacerbate the patients' suffering and result in inadequate care during critical moments. This issue underscores the need for greater awareness and preparedness among emergency medical staff to handle the complexities of SCD, ensuring that patients receive the appropriate care during urgent situations, including the need to ‘jump the line’ when dealing with SCD patients in a pain crisis.

The interviews also highlighted significant resource constraints that affect the care of patients with SCD. Participant 27 (advocate) reported that there are ‘*not enough sickle cell clinics. Not enough time. Not enough providers*.’ This statement reflects a systemic issue where the infrastructure needed to support SCD management is insufficient. Participants highlighted that the shortage of dedicated sickle cell clinics means that patients may have limited access to facilities that are equipped to address and understand their specific needs. Additionally, the lack of sufficient time allowed for patient interactions with providers indicates that even when clinics are available, they may be overwhelmed with patient demands, leading to reduced quality of care. These resource limitations can create barriers to accessing timely and effective treatment, making it more challenging for patients to manage their condition and maintain their health.

Together, these findings illustrate the multifaceted problems in the current care system for SCD. Addressing these issues requires a concerted effort to increase the number of specialised care providers, improve the responsiveness of emergency care and expand resources to ensure that patients have access to the necessary care and support.

#### 3.2.3 Theme 3: Barriers to Coping

Unaddressed past experiences with unresponsive care and overwhelmingly complex psychosocial needs of patients and caregivers impact the patients' receptivity to care, and perceived need for care constituted the final category of barriers identified by participants. This theme highlights the diverse challenges sickle cell patients face when accessing care. These challenges include difficulties in accessing psychological support, a lack of effective coping mechanisms and the profound impact of chronic pain and fatigue on daily life. Participants emphasised how these struggles exacerbate the burden of living with a chronic illness, complicating disease management and diminishing overall quality of life. Participants highlighted the critical importance of addressing these interconnected issues, including fostering access to community and peer support networks.

One participant highlighted the importance of additional support, noting that,

‘Socio‐economic status of some of our patients, you know even additional resources such as food stamps or and you know, access to social security checks might be important, especially, [if] they are not able to work.’

Participant 21, HCP

This underscores the necessity for patients to have access to financial and social support systems that can help them manage their condition and maintain their quality of life when they are unable to work. Participants emphasised how access to such resources is crucial for ensuring that patients have the means to meet their basic needs, which can significantly impact their overall health and well‐being. This, in turn, makes them much more vulnerable to pain crises, leading to increased admissions, and if not dealt with fully, including preventively with follow‐up systems, will lead to readmissions.

Discrimination by HCPs is another significant issue reported. One participant remarked,

‘You know our patients have had discrimination from other providers that do not understand the disease.’

Participant 21, HCP

Patients with SCD often face negative attitudes and a lack of understanding from some HCPs, which can negatively affect the quality of care they receive. Addressing these discriminatory practices and ensuring that providers have a thorough understanding of SCD are important steps in improving patient outcomes and fostering a more supportive healthcare environment.

The need to address social determinants of health was also emphasised. A participant suggested that

‘Addressing the Social Determinants of Health would be very helpful to take care of this population now[that] has been neglected for a very long time and talking about access to food, access to social security checks. access to employment, if they would want to be employed and talking about you know any support system that would have appears support for them so that they can unify their thoughts and you know common understanding of the disease.’

Participant 21, HCP

This highlights the broad range of social factors that impact the health of patients with SCD, including access to basic needs, employment opportunities and support systems. While needed for all patients, such basics are especially needed for SCD patients, who often struggle to stay in school while seeking an education and/or keep a job, due to crises, and thus struggle to gain financial stability. Yet, it is well known that by addressing these social determinants, there is the potential to improve the overall quality of care and support available to this population.

The importance of strong community support was also identified as a crucial factor in patient well‐being. A participant noted, ‘*I think patients having strong community groups is very crucial so making sure that they're like I mentioned, you know connected to other sickle cell patients but also just connected to people, I mean good relationships and friendships*’ (Participant 20, HCP). Participants identified that fostering connections among patients with SCD, as well as encouraging broader social relationships, play a significant role in providing emotional support and enhancing overall health.

The insights from the interviews emphasise the critical need for comprehensive support that addresses socio‐economic factors, combats discrimination and fosters community connections. Focusing on these areas offers significant potential to enhance the support system and improve health outcomes for patients with SCD. The identified themes and descriptive quotes are summarised in Table 2.

**Table 2:** Summary of themes and illustrative quotes.

Theme 1: Structural barriers, the manifestation of systemic racism, affecting access to care and healthcare experiences
**Quotes:** ‘*… now you're talking specialists needing to be involved with hematology and it's clear that there's just not a lot of reimbursement to even cover the cost of it from that angle.’—*(Participant 29, administrator). ‘*So, it's a huge financial hit as well.’—*(Participant 29, administrator). ‘*There's a lot of stigma with the disease, even as a resident when people still didn't know that I had the disease. I would hear very negative comments coming from other residents or other physicians and attendings themselves.’—*(Participant 16, patient). ‘*There is a stigma associated with us being African‐American and as adults when we walk in the door, when they walk in the door, both categories, they're perceived with the prejudice. With the historic prejudice.’—*(Participant 26, advocate). ‘*They try to treat all [people with SCD/T] the same, but that's not how it works*’—(Participant 17, patient).
**Theme 2: Healthcare provider‐related barriers—the lack of knowledge, understanding and compassion from providers impacts both the availability and quality of care.**
**Quotes:** ‘*Some hematologists don't even deal with sickle cell*’—(Participant 26, advocate). ‘*You know our patients have had discrimination from other providers that do not understand the disease.’—*(Participant 21, HCP). ‘*Not enough sickle cell clinics. Not enough time. Not enough providers. None of that. We don't have any of that really.’—*(Participant 27, advocate).
**Theme 3: Barriers to coping—unaddressed psychosocial needs of patients and caregivers impact the patients' receptivity to care and perceived need for care.**
**Quotes:** ‘*Socio economic status of some of our patients, you know even additional resources such as food stamps or and you know, access to social security. Checks might be important, especially, they are not able to work, and those are the Community resources that I'm thinking top my head right now.’—*(Participant 21, HCP). ‘*You know our patients have had discrimination from other providers that do not understand the disease.’—*(Participant 21, HCP). ‘*Address the Social determinants of health would be very helpful to take care of this population now has been neglected for a very long time and talking about access to food, access to social security checks. access to employment, if they would want to be employed and talking about you know any support system that would have appears support for them so that they can unify their thoughts and you know common understanding of the disease.’—*(Participant 21, HCP).

## 4 Discussion

Our research highlights the broad spectrum of challenges faced by adults with SCD, uncovering nuanced perspectives within healthcare. Our thematic analysis revealed that patients and HCPs often share similar views about the hurdles, including discrimination, encountered by patients with SCD, while administrators tended to concentrate on the systemic financial burdens impacting SCD healthcare delivery in terms of continuity and quality of care. These findings must be interpreted within the context of the US healthcare system, where access to care is widely fragmented and heavily reliant on insurance coverage [23, 24]. Unlike many countries with universal health care systems, the United States has a mixed healthcare model in which insurance is linked to employment or public programmes like Medicaid or Medicare [25]. Many Individuals with SCD rely on Medicaid, a public insurance programme that varies by state and typically offers lower reimbursement rates to providers compared to private insurance, contributing to reduced provider incentives and access disparities.

It is important to highlight the structural financial barriers that health systems create when caring for patients with SCD, as these barriers contribute to access disparities. Healthcare reimbursement in the United States incentivises shorter hospital stays and minimal use of specialists, even when ongoing specialty care could reduce long‐term costs [26]. SCD care, in particular, is costly due to frequent hospitalisations, pain crises and the need for specialised management [27, 28, 29]. The paradox, however, is that without upfront investment in comprehensive specialty care, patients experience more frequent emergency room visits, longer hospitalisations and higher rates of readmission—ultimately increasing overall healthcare costs [30]. While patients with SCD may be eligible for supplemental insurance coverage, securing such coverage requires an annual re‐application process that places an undue burden on individuals already navigating a complex chronic illness; additionally, this supplemental coverage may not cover [30]. Administratively, this still results in inadequate reimbursement, reinforcing the economic disincentives for medical institutions to invest in long‐term, high‐quality SCD care [31]. Given the biologic course of SCD, readmissions should not be counted toward the statistics for financial penalties. Cancer patients also have high unplanned readmission rates, but because this is regarded as part of the biologic course of the disease, no penalty is incurred [32].

Among the outstanding challenges to caring for adults with SCD, we identified multifaceted financial barriers that impact access to medications, specialised healthcare services and essential support mechanisms. The financial burden of SCD to patients is alarming, even for those who have commercial health insurance coverage [33]. These financial barriers not only hinder patients' adherence to prescribed treatment plans but also exacerbate healthcare disparities among adults with SCD [3]. Resulting symptom exacerbation further impacts adherence, coping and quality of life, creating a vicious cycle of worsened outcomes [17, 34, 35].

Clinical guidelines and protocols are intended to promote the use of evidence‐based healthcare, improve safety and efficacy, and ensure consistent quality of care [36]. However, participants expressed concerns about too rigid and uniform treatment plans rather than individualised care plans that help address the needs of a wide range of types of patients, compounded by a discriminatory healthcare system. While guidelines are helpful, if they are not widely understood, are not followed consistently and implemented with needed flexibility across organisations, HCPs and others, they can backfire and result in poorer outcomes. This finding is consistent with the literature indicating significant SCD health inequities associated with institutional racism and poor implementation of guidelines, further decreasing the quality of care [4, 6]. Perceived racial bias and health‐related stigma have been shown to negatively impact physical, mental, social and spiritual health among patients with SCD [4, 35].

Additionally, funding and reimbursement challenges limit the scope of services that HCPs and healthcare institutions can provide, worsening access to care and deepening inequities in health outcomes for this vulnerable group. When care is inadequate, the frequency of vaso‐occlusive episodes and their complications increases, resulting in increased healthcare costs [37]. It is also important to highlight the need for genetic counselling to help patients make family planning decisions and offer mental health services. When care inadequately addresses common complications of chronic illnesses, such as depression, care utilisation and costs of care are again significantly increased [35, 38].

Adopting a different model of care, such as creating a medical family home specific to patients with SCD, may help address the barriers to care identified. The Classic Comprehensive model includes a dedicated clinical space and dedicated clinical staff. Care under this model is led by a sickle cell specialist and supported by advanced practice providers. The Specialized Medical Home model of care is similar to the classic model in that it utilises a dedicated clinical space and staff; however, the care is delivered in a more collaborative manner that allows patients flexibility to maintain primary care outside of the medical home and still receive more specialised care at the specialty medical home affiliated [18]. Two additional models to consider include Embedded Care and the Hub and Spoke Model. Both of these models utilise shared clinic space and staff. The Embedded Care model typically involves the integration of sickle cell care into an existing clinic structure, such as a cancer centre. The Hub and Spoke model utilised a primary location as its main location of SCD care which includes all comprehensive services (the Hub) while closely collaborating satellite clinics (spokes) provide limited services in areas further from the hub. This model is typically utilised in rural areas, such as the area for which we conducted the study, where many patients live more rurally due to the lower costs of living.

While this paper focuses on the barriers to care experienced by individuals with SCD, we must also acknowledge their strength. Our patients carry on with life, despite the challenges they face day‐in and day‐out. As HCPs and scientists, we owe it to this uniquely vulnerable population to do everything we can to alleviate their suffering and reduce health disparities. This includes addressing all aspects of health (physical, mental, social and spiritual) in the medical model chosen. While SCD management guidelines focus on addressing the complex physical health requirements, the other domains of health are all too often neglected [13]. Yet having strong community support was identified by our participants as an area of strength, which, when supported, could further augment overall health and well‐being [16, 17].

The findings of this study must be considered with acknowledgement of certain limitations. Purposive sampling, while ensuring participants have the requisite knowledge and experiences to share pertaining to the phenomenon of interest, increases the risk of confirmatory bias [39]. However, credibility and reliability are ensured by the use of triangulation and member checking. Triangulation (which we carefully implemented) involves using multiple data sources to help corroborate findings, thus increasing trustworthiness and reducing the likelihood of bias. Member checking with participants, which we did in our feedback focus group, is a method of verifying the accuracy and authenticity of interpretations [21]. Additionally, while the findings may not be applicable to all patients, the rigour of the research enhancing transferability to SCD populations in other regions is likely [39].

The qualitative findings of this study add an important, nuanced understanding to the literature on barriers to care for the sickle cell community, particularly with the addition of healthcare administration and payer group perspectives. They also speak to the complexity and conundrum of establishing an SCD patient medical home, which is a steep investment, but much needed to break the readmission cycle of cost and human suffering. As part of such a medical home, to address the challenges faced by adults with SCD, healthcare systems must enhance training for providers (e.g. implicit bias, cultural competency and adherence to evidence‐based guidelines) while advocating for increased funding to reduce financial barriers to care. Policies should target systematic discrimination and promote equitable resource allocation, while expanding access to critical services like mental health counselling and genetic counselling. Additionally, fostering patient‐centred communication, conducting focused research on inequities and building community partnerships to facilitate continued care for ongoing optimal functioning can help improve outcomes and reduce disparities. Public awareness and advocacy efforts may also be useful in prioritising SCD as a public health issue.

Recommendations for moving forward include updating clinical practice guidelines and training to address these wider issues and involving patients as leaders in the process. Patients have knowledge and expertise that would benefit both clinical and financial administrators in making much‐needed changes. Acknowledging racial bias, lack of personalised medicine and treating patients with SCD with human dignity (not them as economic burdens to be dealt) is a place to start. The field of medicine is innovative and rapidly changing with technology such as gene therapy. Isn't it time for the culture of hospitals and the funding context to change too?

## 5 Conclusions

Our research underscores the need for a comprehensive and interdisciplinary approach to SCD care, a system that involves patients, HCPs, administrators and the broader community, to overcome their complex barriers to better care. We advocate for systemic changes to ensure that individuals with SCD/T are afforded dignified, empathetic and high‐quality care. By tackling these issues, healthcare systems can move closer to delivering equitable care and improving the lives of those affected by SCD.

## Author Contributions

**Chanell Grismore:** conceptualisation, methodology, formal analysis, data curation, writing – original draft, project administration. **Lisa R. Roberts:** conceptualisation, methodology, formal analysis, writing – original draft, writing – review and editing. **Zephon D. Lister:** validation, data curation, visualisation. **Akshat Jain:** visualisation. **Julio Silvestre:** visualisation. **Ja'Nece Dickerson:** validation, data curation, visualisation. **Susanne B. Montgomery:** validation, investigation, resources, data curation, writing – review and editing, supervision.

## Disclosure

This content and conclusions are those of the authors and should not be construed as the official position or policy of, nor should any endorsement be inferred by, HRSA, HHS or the U.S. government.

## Ethics Statement

The study was conducted in accordance with the Declaration of Helsinki and approved by the Institutional Review Board of Loma Linda University (#5190109, 28 December 2021).

## Conflicts of Interest

The authors declare no conflicts of interest.

## Data Availability

The data presented in this study are available upon request from the corresponding author due to privacy concerns for participants.
